# Imperfect diet choice reduces the performance of a predatory mite

**DOI:** 10.1007/s00442-023-05359-0

**Published:** 2023-03-22

**Authors:** Felipe Lemos, Sabina Bajda, Marcus V. A. Duarte, Juan M. Alba, Thomas Van Leeuwen, Angelo Pallini, Maurice W. Sabelis, Arne Janssen

**Affiliations:** 1 Department of Evolutionary and Population Ecology, IBED, University of Amsterdam, Science Park 904, 1098 XH Amsterdam, The Netherlands; 2 Laboratory of Acarology, Department of Entomology, Federal University of Viçosa, 36, Viçosa, MG 570-000 Brazil; 3 Present Address: Ecofit- Bioinsumos, Araxá, MG Brazil; 4 Department of Plants and Crops, Faculty of Bioscience Engineering, Ghent University, Ghent, Belgium; 5 Present Address: R&D Department, Biobest Group NV, Westerlo, Belgium

**Keywords:** Mixed diet, Nutritional ecology, Toxic prey, Phytoseiids, *Tetranychus evansi*

## Abstract

Two mutually unexclusive hypotheses prevail in the theory of nutritional ecology: the balanced diet hypothesis states that consumers feed on different food items because they have complementary nutrient and energy compositions. The toxin-dilution hypothesis poses that consumers feed on different food items to dilute the toxins present in each. Both predict that consumers should not feed on low-quality food when ample high-quality food forming a complete diet is present. We investigated the diet choice of *Phytoseiulus persimilis*, a predatory mite of web-producing spider mites. It can develop and reproduce on single prey species, for example the spider mite *Tetranychus urticae*. A closely related prey, *T. evansi*, is of notorious bad quality for *P. persimilis* and other predator species. We show that juvenile predators feeding on this prey have low survival and do not develop into adults. Adults stop reproducing and have increased mortality when feeding on it. Feeding on a mixed diet of the two prey decreases predator performance, but short-term effects of feeding on the low-quality prey can be partially reversed by subsequently feeding on the high-quality prey. Yet, predators consume low-quality prey in the presence of high-quality prey, which is in disagreement with both hypotheses. We suggest that it is perhaps not the instantaneous reproduction on single prey or mixtures of prey that matters for the fitness of predators, but that it is the overall reproduction by a female and her offspring on an ephemeral prey patch, which may be increased by including inferior prey in their diet.

**Supplementary Information:**

The online version contains supplementary material available at 10.1007/s00442-023-05359-0.

## Introduction

Many predators face the task of selecting their diet from among various prey types and species, and, according to the balanced diet hypothesis, feed on a mixed diet to acquire energy and nutrients needed for growth and development (Pulliam 1975; Bilde and Toft 1994; Raubenheimer and Simpson 1997; Mayntz et al. 2005; Lefcheck et al. 2013). They also need to avoid adverse effects of feeding on toxic prey, which they can do by mixing them with non-toxic prey (the so-called toxin-dilution hypothesis, Freeland and Janzen 1974; Toft and Wise 1999a; Lefcheck et al. 2013). Toft and Wise (1999a) define high-quality prey as those that sustain development and reproduction and result in low mortality. Low-quality prey are then those that fall short in allowing for development, survival and/or reproduction, and this may be because of lack of essential nutrients or because of toxicity. Feeding on such low-quality prey is not expected under natural conditions unless it confers some benefit to the predators (Toft 1995), for example, because they provide nutrients or energy that would be in short supply when feeding on high-quality prey only. This includes feeding on a limited number of low-quality prey to avoid starvation when other prey are scarce (Rickers et al. 2006; Glendinning 2007). Thus, scarcity of high-quality prey could result in selection for adaptation to low-quality prey or other food, hence, towards more generalist feeding habits. Otherwise, adding low-quality food to a high-quality diet usually decreases predator performance (Eubanks and Denno 1999; Toft and Wise 1999a, b; Bilde and Toft 2001; Oelbermann and Scheu 2002; Lefcheck et al. 2013), and is therefore not expected. If toxic prey do not provide scarce nutrients and predators do not innately have an aversion to them, predators can deal with their occurrence in the environment either by learning to avoid them (Fisker and Toft 2004; Glendinning 2007; Nelson et al. 2011) or by becoming tolerant to the toxins (Freeland and Janzen 1974; Glendinning 2007). In contrast to these theoretical expectations, here we present evidence that a predator feeds on a prey species of low quality, even when its high-quality prey is abundant and there is no advantage of feeding on a mixed diet.

We used the well-studied predatory mite species *Phytoseiulus persimilis*, a biological control agent of the two-spotted spider mite (*Tetranychus urticae*) in many agricultural systems (van Lenteren 2012). This spider mite is used here as benchmark high-quality prey. The predator can develop and reproduce perfectly well during multiple generations on a diet of *T. urticae* alone (Stenseth 1979), hence, apparently does not need a mixed diet. *Phytoseiulus persimilis* does not perform well on *Tetranychus evansi*, which is a low-quality prey for several predators (de Moraes and McMurtry 1985, 1986; Escudero and Ferragut 2005; Koller et al. 2007; de Vasconcelos et al. 2008). Thus far, only one predatory mite species, *Phytoseiulus longipes*, has been reported to perform well on it (Furtado et al. 2007; da Silva et al. 2010; Ferrero et al. 2014). Although the two spider mite species are closely related, there are more differences between them than their quality as prey. *Tetranychus urticae* is extremely polyphagous, attacking more than 1150 plant species worldwide (Migeon and Dorkeld 2020). *Tetranychus evansi* is more specialized, occurring on c. 130 plant species, especially solanaceous plants (Migeon and Dorkeld 2020). It has been suggested that *T. evansi* is a low-quality prey because it accumulates or sequesters the toxic secondary plant compounds amply present in their solanaceous host plants (Kennedy 2003; Escudero and Ferragut 2005; Koller et al. 2007; Ferrero et al. 2014). Another difference between the two species is that many strains of *T. urticae* induce defences in their host plants, whereas *T. evansi* suppresses them in several plant species (Kant et al. 2004; Sarmento et al. 2011a; Alba et al. 2015; de Oliveira et al. 2016; Knegt et al. 2020). Lastly, *T. urticae* is found almost everywhere (Migeon and Dorkeld 2020), but *T. evansi* originates from South America and has only recently invaded Africa, Europe and Japan (Navajas et al. 2013), In Europe, it now co-occurs with *T. urticae* and *P. persimilis* (Escudero and Ferragut 2005). Several of these differences in ecology of the two spider mite species have been implicated as being related to their quality as prey. Here, however, we do not investigate the cause of the low quality of *T. evansi* as prey, but concentrate on its consequences for the diet choice and performance of *P. persimilis*.

We first assessed the effects of feeding on low-quality prey and on a mixture of low-quality and high-quality prey on predator survival and development. We also evaluated prey preference and reproduction on the two prey and investigated whether experience with the low-quality prey resulted in the development of aversion towards this prey. Furthermore, we investigated the reversibility of the negative effects of feeding on the low-quality prey on development and reproduction.

## Material and methods

### Organisms

Tomato seeds (*Solanum lycopersicum* var. Santa Clara I-5300) were sown in soil (50% coco peat, 15% white peat, 35% frozen black peat, Jongkind Grond BV, Aalsmeer) in a PVC tray (6 × 12 cells) and plants were transplanted to plastic pots (2 L) with soil 14 days after germination. The rearing units of *T. evansi* and *T. urticae* at the University of Amsterdam were started with specimens from the Laboratory of Acarology, Federal University of Viçosa, Minas Gerais, Brazil, collected in 2002 from infested tomato plants (Sarmento et al. 2011a, b). They were reared on detached tomato leaves kept in plastic trays (30 × 22 cm, 8 cm high) containing water to maintain leaf turgor. These trays were placed inside larger trays (54 × 38 cm, 8.5 cm high) filled with water with detergent (c. 1:50, v/v) to prevent escapes of mites and contamination among populations. The rearing units were maintained in a climate room (25 °C, 70 – 80% relative humidity, 16 h light).

*Phytoseiulus persimilis* was obtained from Koppert (Berkel and Rodenrijs, the Netherlands) and was kept on detached cucumber leaves infested with *T. urticae*, to which it was adapted. From this stock rearing unit, we started a new unit 1 month before the experiments, which was fed with the Brazilian strain of *T. urticae*. This unit was kept in a plastic tray (30 × 22 cm, 8 cm high) inside a larger PVC tray (54 × 38 cm, 8.5 cm high) filled with water with detergent (as above). Two to three tomato leaves infested with spider mites were added to the inner tray every three to four days. The units were maintained in a climate room (as above).

The predators used in our experiments came from cohorts obtained by taking approximately 30 adult females of *P. persimilis* from the new rearing unit and transferring them to tomato leaflets infested with *T. urticae* arranged on wet cotton wool in plastic trays. The females were allowed to feed and oviposit for 24 h, were then removed, and the leaflets with predator eggs and prey were either incubated in a climate room (as above), or predator eggs were used immediately for the experiments. Adult female *P. persimilis* go through a pre-oviposition period of around 1.5 days, during which they increase about 60% in weight and reach a body length of c. 0.6 mm (Sabelis 1981). After 7–9 days, the cohort of predatory mites was adult; the females were allowed to mate and were subsequently used for experiments.

### Juvenile performance

We measured the performance of juvenile predatory mites feeding on eggs of *T. urticae* and *T. evansi*. Predators without food were used as control. The predators develop from eggs to a six-legged larva stage, the only mobile stage that does not require food, to the eight-legged protonymph (Takafuji and Chant 1976; Sabelis 1981). Protonymphs start feeding on spider mite eggs and juveniles and develop into deutonymphs, which develop into adults.

Leaf discs (diameter 24 mm) were cut from leaves of clean tomato plants and arranged on paper tissue soaked with water, positioned on wet foam inside a tray (12.5 by 7.5 cm, 2.5 cm high) filled with tap water. The wet tissue prevented the mites from escaping from the discs. Each leaf disc was infested with 20 adult females of *T. evansi* or *T. urticae*, taken from stock rearing units. The trays with the leaf discs were incubated in a climate room (as above) for 24 h. Subsequently, spider mite females and the web produced by them were removed from leaf discs using a fine paintbrush, leaving their eggs behind. The numbers of eggs of spider mites on each leaf disc were recorded.

One egg from a cohort of *P. persimilis* was transferred to each leaf disc. For logistical reasons, this experiment was performed in three blocks that were repeated in time. As the stock populations of the two spider mites fluctuated differentially through time, the number of replicates varied according to the availability of spider mites and ranged from 18 (*T. urticae* and no food, with 6 replicates in each of three blocks) to 24 (*T. evansi* with 6, 12 and 6 replicates in the three blocks). We observed the development, survival and predation rate of juveniles of *P. persimilis* from egg to adult once a day. The various stages were assessed by checking for exuviae resulting from moulting. Because spider mite eggs take around four days to hatch at 25 °C (Bonato 1999), the leaf discs were replaced with new leaf discs with spider mite eggs of the corresponding treatments every three or four days. At removal, ample numbers of prey eggs were still present. Leaf discs without spider mite eggs (treatment No food) were also replaced.

Survival data were analyzed with a Cox mixed-effects proportional hazards model (package coxme, Therneau 2015) with block as a random factor and food type as factor. Pairwise comparisons among diets were done with the function emmeans from the package with the same name (Lenth 2019). Because the treatment without food did not result in development into adults for obvious reasons, development data were analyzed with the Kaplan–Meier estimates (packages survdif and survminer, Therneau 2020; Kassambara et al. 2020) with prey type as a factor. The average predation rate per day was calculated per juvenile and was log(x + 1) transformed and compared for the two prey species with a linear mixed-effects model (LME, function lme of the package nlme, Pinheiro et al. 2020) with prey as a factor and block as a random factor. All statistics were calculated with R statistical software (R Core Team 2020).

### Prey preference and adult performance on mixed and single diets

After a pre-oviposition period, female *P. persimilis* convert spider mites into predator eggs with an efficiency of c. 70% on a weight basis (Sabelis 1981), hence, reproduction is strongly affected by prey consumption. We, therefore, assessed both oviposition and prey consumption by adult female predators. Tomato leaf discs (as above) were cut from detached leaves so that the main leaf vein divided the discs into two halves with approximately similar areas. Each disc half was infested with 20 adult females of either *T. evansi* or *T. urticae* from the stock rearing units. To prevent spider mites from crossing from one half to the other, a thin thread of wet cotton wool was put along de midrib, contacting the surrounding water so that it was soaked (Pallini et al. 1998). The trays with the leaf discs were incubated in a climate room (conditions as above) for 24 h, adult female spider mites and web were subsequently removed and eggs were counted. The cotton thread was removed from the leaf midrib and an entomological pin was inserted at the centre of the disc, drilling the midrib (Dicke and Dijkman 1992). An adult female *P. persimilis* (8–10 days since egg stage) was carefully placed on the tip of the entomological pin. The predators immediately moved down to the leaf disc and started attacking eggs. Similar leaf discs were prepared for each prey species separately as controls, except that we transferred 20 adult females of the same prey species (40 adult females per disc in total) to both halves of the leaf discs, removed them and their web 24 h later and counted the eggs as above. The numbers of eggs (± s.e.) of both prey species encountered in the mixed prey species treatment and controls were 122.08 (± 2.39) for *T. urticae* and 111.81 (± 3.36) for *T. evansi*. The experiment was done in two blocks in time, with each block having replicates of all treatments. There were 12 replicates of each treatment with 3 replicates of each in the first block and 9 of each in the second block.

The survival, predation and oviposition rates of *P. persimilis* were observed during four consecutive days, after which ample numbers of spider mite eggs were still present. Data of individuals that died or went missing were included in the analysis of predation and oviposition data until the day they were absent. The predation data of predators that received a mixed diet served to analyse prey preference by comparing the numbers of each of the two prey species eaten with an LME with prey species and its interaction with time as fixed factors and each individual predatory mite as a random factor to account for repeated measures. The blocks in which the experiment was performed were initially entered as a second random factor, but proved not significant, so were removed. Contrasts were assessed with the package emmeans (Lenth 2019). Survival of adult predators during the experiment was analyzed with Kaplan–Meier estimates (Kassambara et al. 2020; packages survdif and survminer, Therneau 2020) with the diet as a factor. To assess whether there was an effect of the numbers of *T. evansi* consumed on predator survival, we analyzed the survival of individuals feeding on a mixed diet or a diet of *T. evansi* with a GLM with a binomial error distribution (logit link) with the average daily intake of *T. evansi* eggs as factor.

### Reversibility of diet effects

Because the previous experiments showed negative effects of feeding on *T. evansi* eggs on juvenile and adult performance, we investigated whether these effects were reversible, in which case the negative effects of temporarily feeding on low-quality prey may be limited. For juveniles, leaf discs were prepared as above, and larvae of *P. persimilis* (one day old) were each transferred to a leaf disc, containing either eggs of *T. evansi*, eggs of *T. urticae* or no food. Survival and development of the juvenile predators were assessed during two consecutive days. After these two days, the effects of the different diets were apparent, and we transferred the juveniles to new leaf discs with eggs of *T. urticae*, prepared as above, to study the reversibility of the diet effects. Subsequently, the development and survival of the juveniles of *P. persimilis* were evaluated daily until they reached adulthood or died. We registered the numbers of eggs killed by the juvenile predators throughout the experiment. There were 12 individuals for each treatment. Survival and development until adulthood were analyzed with a Cox proportional hazards model as above.

For adult performance, leaf discs were cut from tomato leaves and arranged in plastic trays as described above, and 20 adult female spider mites (*T. urticae* or *T. evansi*) from the stock colonies were used to infest leaf discs separately. Further preparation of the discs was as above. All adult female predatory mites (age as above) were offered leaf discs with eggs of *T. urticae* for the first two days to verify that they oviposited. Subsequently, they were transferred to new leaf discs with either eggs of *T. evansi*, eggs of *T. urticae* or no food and were kept on these leaf discs for two consecutive days, after which they were transferred to new leaf discs with eggs of *T. urticae*. Survival, the numbers of eggs they produced, and the numbers of prey eggs killed were assessed daily. Survival data of females that were alive for at least three days were analyzed with a Cox proportional hazards model in R (Therneau 2020). This resulted in 19, 21 and 23 individuals for the treatments with *T. evansi*, without food and with *T. urticae,* respectively. Oviposition of predators that did not survive until the fourth day of the experiment was excluded from the analysis, resulting in 10, 11 and 18 individuals for the treatments with *T. evansi*, without food, and with *T. urticae*, respectively. The remaining data were analyzed with a linear mixed-effects model (Pinheiro et al. 2020) with individuals as a random factor and treatment and day as factors. To check whether the predators receiving the three treatments did not differ from each other before treatments, we first analyzed the data of the first two days for all three groups together. Subsequently, oviposition data of the third and the fourth day were analyzed for the two groups that received prey, and for all three groups for the last two days.

## Results

### Juvenile performance

**Fig. 1:**
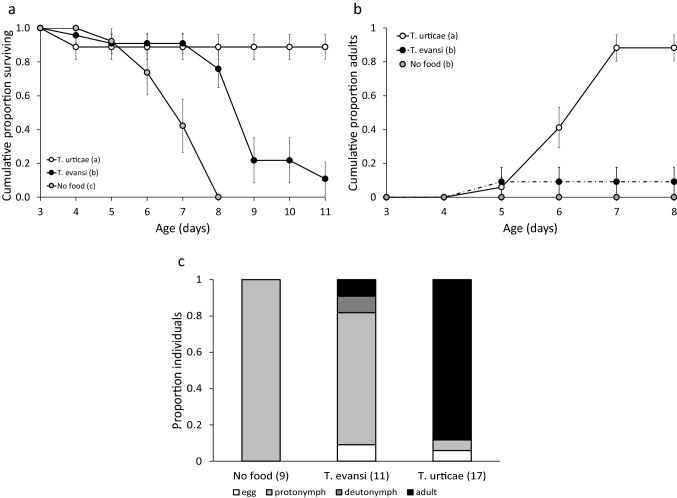
The performance of juvenile *Phytoseiulus persimilis* on diets consisting of eggs of *T. urticae* or *T. evansi* on leaf discs of bean, cucumber or tomato. **a**. The cumulative proportion (± s.e.) of juveniles surviving from the egg stage to adulthood per prey species as a function of individual age. Letters next to the legend give significance among diets. **b**. The cumulative proportions (± s.e.) of juveniles that became adult as a function of age of the individuals. Letters next to the legend give significance among diets. **c.** The final stages reached by the juveniles as function of their diet, shown as a proportion of the initial numbers of individuals

Immature survival varied significantly with diet (Fig. 1a, Cox mixed-effects model: *Chi*^*2*^ = 27.5, *d.f.* = 2, *P* < 0.0001). Immature *P. persimilis* survived significantly better on a diet of eggs of *T. urticae* than on eggs of *T. evansi* and without food. Diet also significantly affected the cumulative proportions of juveniles that developed into adults through time (Fig. 1b, Mantel–Heanszel test of Kaplan–Meier survival estimates: *Chi*^*2*^ = 32.3, *d.f.* = 2, *P* < 0.0001). As expected, juveniles did not develop into adults without prey. Most juveniles became adults when feeding on eggs of *T. urticae*, and only one juvenile became adult on a diet of *T. evansi* (Fig. 1b). The developmental rate was significantly higher on a diet of *T. urticae* than on the other diets (Fig. 1b). Larvae raised without food that did not go missing during the experiment developed into the next stage (protonymphs) (Fig. 1c). On a diet of *T. evansi*, most individuals died as protonymphs, a few developed into deutonymphs, one developed into an adult and one died as egg (Fig. 1c). On a diet of *T. urticae*, most individuals developed into adults, but one died as egg and one as protonymph (Fig. 1c).

All protonymps and deutonymphs consumed eggs of the species present, but juveniles on a diet of *T. evansi* consumed on average 4.5 (s.e. ± 0.5) eggs and those on a diet of *T. urticae* only 2.4 (± 0.2) eggs, and this difference was significant (LME: likelihood ratio = 11.9, *d.f.* = 1, *P* = 0.0006). This shows that the lack of development on a diet of eggs of *T. evansi* was not caused by lack of finding prey eggs.

### Prey preference and adult performance on mixed and single diets

**Fig. 2:**
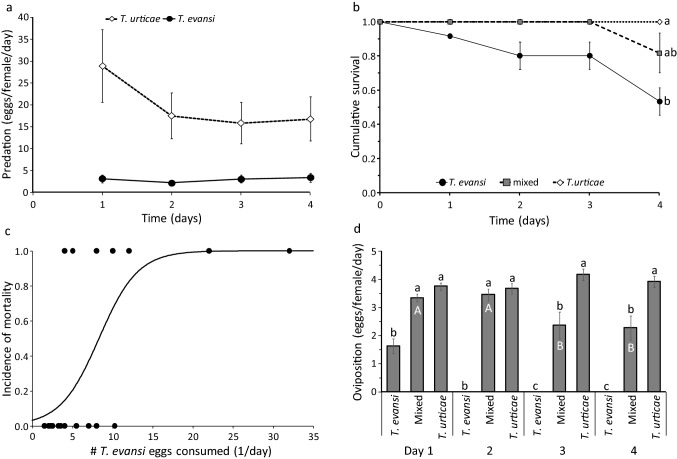
Performance of adult female *P. persimilis* on diets of eggs of *T. urticae*, *T. evansi* or on a mixture of the two. **a**. Average predation (± s.e.) of eggs of the two species during 4 days by predators feeding on a mixed diet. **b**. The cumulative proportion of adults (± s.e.) surviving during the four days of the experiment. Letters next to the curves give significance among diets. **c**. Incidence of mortality as a function of the average number of *T. evansi* eggs consumed per day. The curve is fitted with a GLM with a binomial error distribution. **d.** Average oviposition (± s.e.) by females feeding on these diets during 4 days. Vertical text along horizontal axis gives diet. Different letters above bars give significance of difference among diets per day, white capital letters within bars give significance of difference among days per diet (contrasts after LME, *P* < 0.05)

All adult predators receiving a mixed diet consumed *T. evansi* eggs, but they consumed significantly more eggs of *T. urticae* than of *T. evansi* (Fig. 2a mixed treatment, LME: likelihood ratio = 85.4, *d.f.* = 1, *P* < 0.001). This preference did not change significantly throughout the experiment (interaction of prey species with time, likelihood ratio 2.59, d.f. = 1, *P* = 0.107), showing that the predators did not learn to avoid feeding on *T. evansi*.

The survival of adult predators differed significantly among diets (Mantel–Heanszel test of Kaplan–Meier survival estimates: *Chi*^*2*^ = 16.1, *d.f.* = 2, *P* = 0.0003), with a diet of *T. evansi* alone resulting in significantly lower survival than the other two diets (Fig. 2b). For those predators that fed on eggs of *T. evansi* (diet *T. evansi* and mixed diet), mortality increased significantly with the numbers of *T. evansi* eggs consumed (Fig. 2c, GLM: *Chi*^*2*^ = 16.9, *d.f.* = 1, *P* = 0.001).

Predators on a diet of *T. evansi* alone produced significantly fewer eggs than predators with the two other diets throughout the experiment; their oviposition on the first day was mainly based on the food ingested on the day before (Sabelis 1990) and they stopped ovipositing after the first day (Fig. 2d). Predators on a mixed diet produced similar numbers of eggs as predators on a diet of *T. urticae* during the first two days, but subsequently produced significantly fewer eggs. Together, this resulted in a significant effect of the interaction of diet with time (LME: likelihood ratio = 15.9, *d.f.* = 2, *P* = 0.0004). The differences in oviposition rates among diets were largely a reflection of the differences in predation rates (Supplementary Material S1).

Together, these results show that eggs of *T. evansi* are indeed of low quality for adult predators, causing increased mortality and decreased oviposition. The predators preferred the high-quality prey, but they did not show signs of completely avoiding feeding on low-quality prey in the presence of high-quality prey.

### Reversibility of diet effects

**Fig. 3:**
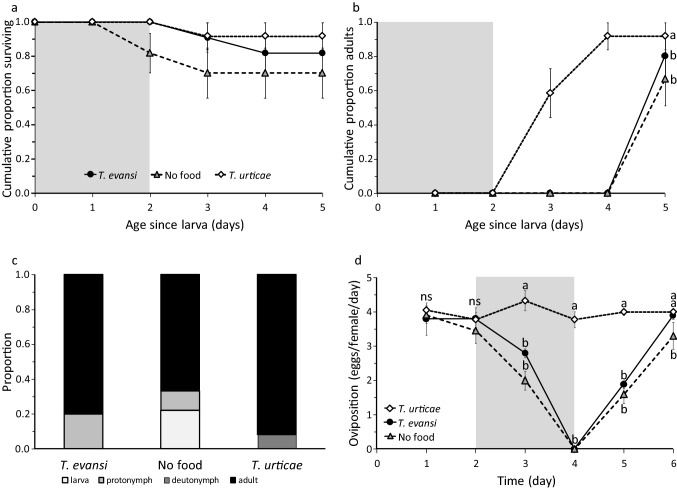
The reversibility of the effect of a diet of *T. evansi* eggs in juvenile (**a**–**c**) and adult (**d**) predators. **a**–**c**: One day old larvae, which soon developed into the next stage, were kept on a diet of eggs of *T. urticae*, *T. evansi* or without food for two days (indicated by the grey background) and were subsequently reared to adulthood or until they died on a diet of *T. urticae* eggs (white background). **a**. Cumulative proportions (± s.e.) of juveniles on the three different diets surviving through time. **b**. Cumulative proportions (± s.e.) of juveniles reaching the adult stage through time. Letters next to final points indicate significance of difference in development among diets. **c**. The final stages reached by the juveniles as function of their diet, shown as a proportion of the initial numbers of individuals. **d**. Young adult females were offered a diet of *T. urticae* eggs during the first two days, then received no food, a diet of *T. urticae* eggs or a diet of *T. evansi* eggs during two days (grey background), and were then returned to a diet of *T. urticae*. Shown are average oviposition rates (± s.e.) of individuals on the three different diets through time. Letters near averages show significance of difference among treatments per day (contrasts after LME, *P* < 0.05, *ns* not significant)

Because the previous experiments showed a negative effect of consuming *T. evansi* eggs on the performance of *P. persimilis*, we investigated whether these effects were temporary or permanent. The juvenile survival of predators that were reared on different diets during the first two days since larva and then on eggs of *T. urticae* did not differ significantly among groups receiving different diets (Fig. 3a, Cox proportional hazards: Likelihood ratio test = 2.09, *d.f.* = 2, *P* = 0.4). The survival of individuals that did not receive food for 2 days was slightly lower than that of individuals that received eggs of *T. urticae* or of *T. evansi* (Fig. 3a), but the majority of these individuals survived the two days without food.

The diet during the first two days of the protonymph stage significantly affected the developmental rate (Fig. 3b, Mantel–Heanszel test of Kaplan–Meier survival estimates: *Chi*^*2*^ = 29.4, *d.f.* = 2, *P* < 0.0001). Immatures that had fed continuously on *T. urticae* developed significantly faster into adults than immatures on the other diets (Fig. 3b). A few individuals that did not receive food during the first two days died as larva, some individuals without food and with *T. evansi* died as protonymph, some individuals feeding on *T. urticae* died as deutonymph, but the majority of individuals of this last group developed into adults (Fig. 3c). Juveniles offered a diet of *T. evansi* eggs consumed many more eggs than those with a diet of *T. urticae* eggs, but after having been offered *T. urticae* eggs, predation did not differ among the three treatment groups (Supp. Mat. S2, Fig. S2a).

Subsequently, we assessed the reversibility of the negative effects of feeding on *T. evansi* eggs for adult female predators. Survival of adult predators that received *T. evansi* (47.4%) or no food (47.6%) for two days was lower than that of predators on a diet of *T. urticae* (60.9%), but this difference was not significant (Cox proportional hazards: Likelihood ratio test = 2.05, *d.f.* = 2, *P* = 0.4). The numbers of predator eggs produced during the first two days, when all of them received eggs of *T. urticae*, did not differ significantly among groups during this period (Fig. 3d, Likelihood ratio = 1.59, *d.f.* = 2, *P* = 0.71). During the second period, the oviposition rates of the group of predators without food and that receiving eggs of *T. evansi* dropped significantly relative to that of the group with eggs of *T. urticae*, and the former two groups did not oviposit on the fourth day (Fig. 3d, LME, interaction treatment with time: likelihood ratio = 43.0, *d.f.* = 2, *P* < 0.0001). The oviposition on the first day after changing diet was again partially affected by the diet of the day before (Sabelis 1990). In the last period, all predators received eggs of *T. urticae* for another two days, and oviposition differed significantly among the three groups (Fig. 3d, LME, interaction of treatment with time: Likelihood ratio = 38.1, *d.f.* = 2, *P* < 0.0001). The groups that had been without food or with *T. evansi* started ovipositing again on day 5, and the oviposition rates of the groups that had fed on *T. evansi* or *T. urticae* did not differ from each other on day six (Fig. 3d). The predators that received *T. evansi* during two days consumed low numbers of this prey, but upon being switched back to a diet of *T. urticae* eggs, they resumed predation within one day, as did the predators that did not receive food during this period (Supp. Mat. S2, Fig. S2b). Together, these data show that the negative effects of a diet of *T. evansi* eggs on the performance of juvenile and adult predators are reversible and comparable to having no food.

## Discussion

In summary, we show that feeding on *T. evansi* negatively affected the performance of the predatory mite *P. persimilis*. The negative effects were reversible when predators were feeding on the low-quality prey for short periods. We furthermore show that the adult predators did have a preference for *T. urticae*, the high-quality prey, but that they did not avoid feeding on the low-quality prey when ample high-quality prey was present.

Our results largely corroborate results by De Moraes and McMurtry (1985; 1986), who also found reduced oviposition and survival of *P. persimilis* on a diet of *T. evansi*. They observed that the predators often pierced the chorion of *T. evansi* eggs but apparently did not feed on them. The same may have occurred in our experiments, so the numbers of eggs reported as preyed here might be a combination of the actual numbers preyed and killed but not fed upon. It can be argued that this piercing of eggs would cost time and energy, resulting in the observed reduced performance of the mites when offered a diet with *T. evansi*. *Phytoseiulus persimilis* is mainly limited by the rate of digestion of prey and not by their encounter rate or the time spent handling prey (Sabelis 1990). If predators on a mixed diet would only have fed on the eggs of *T. urticae*, they would not have lost much time with piercing and rejecting eggs of *T. evansi*, so their performance on a mixed diet would not be significantly worse than on a diet of *T. urticae* only*.* We, therefore, conclude that the reduced performance on a mixed diet was caused by the ingestion of some toxic or digestibility-reducing factor present in the eggs of *T. evansi*.

The definition of high-quality prey by Toft and Wise (1999a) as prey that sustain development and reproduction and result in low mortality certainly applies to a diet of *T. urticae*. The strain of *P. persimilis* used here has been reared exclusively on this prey species for a long time, which may also have led to more adaptation to it. Toft and Wise (1999a) define toxic prey as one that causes higher mortality rates than in controls without food. According to this definition, *T. evansi* does not qualify as toxic for juvenile *P. persimilis*, as these showed a higher survival and some development on a diet of *T. evansi* than without food (Fig. 1a). However, adult predatory mites feeding on *T. evansi* had lower survival than those feeding on other diets and mortality increased with increasing numbers of *T. evansi* eggs consumed (Fig. 2b, c). Although there was no control without food included in this experiment, adult *P. persimilis* are known to survive for at least eight days without food as long as free water is available or when humidity is high (Bernstein 1983; Gaede 1992). Given that water was freely available during the experiments presented here, the lack of food would not have resulted in increased mortality during the experiments described here. Furthermore, the predators that died during the four days of the experiment had, on average, consumed more eggs of *T. evansi* per day than the mites that survived. We, therefore, conclude that *T. evansi* is, at least to some level, toxic to adult *P. persimilis*.

It has been suggested that predators feed on low-quality prey because they do not recognize them as such, but this then begs the question of why they do not. Predators can develop an aversion to toxic prey by learning the association between the harmful effects of the food and other compounds in the food, such as taste (Gelperin 1968; Berenbaum and Miliczky 1984; Fisker and Toft 2004; Rickers et al. 2006; Glendinning 2007; Nelson et al. 2011). In the case of the predator studied here, it is clear that the adult predators preferentially feed on the high-quality prey from the first day that they are offered a mixed diet (Fig. 2a), showing that they can discriminate between the two prey species. Yet, they persist in feeding on the low-quality prey to such an extent that it reduces their performance, at least during the experiments. Feeding on *T. evansi* for longer periods causes irreversible losses in fitness in juveniles (Fig. 1) and adults (Fig. 2), but when the predators fed on the low-quality prey for a period of only a few days, the negative effects could be partially reversed by feeding on high-quality prey afterwards (Fig. 3). For example, almost no juveniles became adults when feeding exclusively on *T. evansi* eggs (Fig. 1a, b), but most did so when feeding on *T. evansi* for a limited period (Fig. 3b, c). Although adult females can survive without food for at least 8 days when they have access to water (Bernstein 1983; Gaede 1992), they will stop ovipositing in the absence of prey. When food becomes available again, they can resume reproduction at the same level as before starvation (Sabelis 1981), and this is what was observed here for females without food and females feeding on *T. evansi*. Hence, avoiding feeding on *T. evansi* may not confer large fitness advantages initially, but will do so after longer periods. Perhaps the predators, therefore, need longer exposure to a mixture of high- and low-quality prey to develop an aversion. *Phytoseiulus persimilis*, however, is known to develop aversion or lack of attraction towards volatiles based on experience in one day (Drukker et al. 2000; de Boer et al. 2005; Zhang et al. 2022), and we suggest that similar learning capacities are present with respect to a combination of tactile and contact-chemical cues, possibly also combined with volatile cues. We, therefore, suspect that there is another reason for the sustained feeding on the low-quality prey, which will be discussed below.

Mixing the toxic prey with high-quality prey resulted in less severe effects on predator performance than feeding exclusively on the low-quality prey, which is in agreement with the toxin-dilution hypothesis. Such effects were found for toxic prey in several other studies (Toft 1995), but there seems to be no advantage of adding the low-quality prey to a diet of high-quality prey (Eubanks and Denno 1999; Toft and Wise 1999a, b; Bilde and Toft 2001; Oelbermann and Scheu 2002; Nielsen et al. 2002). Nevertheless, the predators in our experiments consumed *T. evansi* persistently in the presence of ample high-quality prey, as was also reported in other studies (De Moraes and McMurtry 1986; Snyder et al. 2000; Fisker and Toft 2004; Stamp and Meyerhoefer 2004; Rickers et al. 2006; Hinkelman and Tenhumberg 2013). The balanced diet hypothesis predicts that the performance of the predators on a mixed diet is better than on the single diets (Bilde and Toft 1994; Raubenheimer and Simpson 1997; Lefcheck et al. 2013), which was clearly not the case here. The lack of agreement with these two main hypotheses calls for further hypotheses on diet mixing.

Besides developing an aversion to low-quality food, another way of dealing with it is to ingest small quantities to induce detoxification systems or through selection for intestinal micro-organisms that can help in detoxification (Freeland and Janzen 1974; Nielsen et al. 2000; Fisker and Toft 2004), and perhaps this is what predators were doing in our experiments. As Freeland and Janzen (1974) suggest, such low-quality foods should be ingested with extreme caution, advice that is apparently lost on *P. persimilis*, which suffered consequences of ingesting low-quality food without reducing its intake over subsequent days. Perhaps longer-term exposure of the predator to mixtures of the two prey would result in either increased tolerance to the adverse effects of the low-quality prey or to increased aversion to it.

In their review of the effects of mixed diets, Lefcheck et al. (2013) argue that diet choice depends not only on prey quality, but also on prey densities and the risk of competition and predation. Furthermore, it is perhaps not the instantaneous oviposition rate on certain mixtures of prey or single prey that matters, but the total number of offspring and grand-offspring that is produced throughout the existence of a prey patch, especially for predators that spend several generations on one ephemeral patch. For example, Venzon et al. (2002) suggested that a predator’s prey choice was not determined by prey quality, but patch quality, i.e., the total number of offspring produced in a prey patch throughout its existence. The interaction between predatory mites and spider mites on a patch, consisting of a plant or group of neighbouring plants, typically lasts for several generations (Sabelis et al. 2002), and predators could perhaps adapt to toxins or digestibility reducers present in the low-quality prey in this period. Furthermore, consuming low-quality prey instead of high-quality prey may prolong the total interaction time of the predators and prey on a patch, resulting in the production of higher numbers of dispersing offspring (Pels and Sabelis 1999; Revynthi et al. 2018).

Another potential explanation for the persistent feeding of predators on low-quality prey, even in the presence of abundant high-quality prey, is that predators may often be food limited under natural conditions and, therefore, need to feed on these prey to survive adverse periods (Toft and Wise 1999a, b; Bilde and Toft 2001). In this case, feeding continuously on this low-quality prey perhaps induces the adaptation to this food, and may also result in increased performance under natural conditions in the long run. Contradicting this idea somewhat is the finding that exposure of *P. persimilis* to sublethal doses of acaricides did not seem to induce detoxification genes (Bajda et al. 2022), suggesting that such adaptation through detoxification may not readily occur in this predatory mite. Moreover, another, closely related predatory mite species, *P. macropilis*, co-occurs with *T. evansi* on tomato plants in Brazil, yet can still not reproduce when feeding on it (de Moraes and McMurtry 1985; Rosa et al. 2005), so it remains to be seen if predators can easily adapt to this prey species. Nevertheless, studies of mixed diets should consider testing the effects of diets under natural conditions when laboratory experiments show that they are suboptimal (Lefcheck et al. 2013).

## Supplementary Information

Below is the link to the electronic supplementary material.

Supplementary file 1: (DOCX 70 KB)

## Data Availability

Upon publication of the ms, data will be made available on UvA/AUAS figshare.
