# Supplementary material for: Imperfect diet choice reduces the performance of a predatory mite
Source: Oecologia. 2023 Mar 22;201(4):929–39. doi: 10.1007/s00442-023-05359-0 (PMC10113300; doi:10.1007/s00442-023-05359-0)
Supplement: Supplementary file 1 — (DOCX 70 KB) [file 442_2023_5359_MOESM1_ESM.docx]

**Supporting information for**

**Imperfect diet choice reduces the performance of a predatory mite**

Felipe Lemos^1,2,3^, Sabina Bajda^4^, Marcus V. A. Duarte^1,2,5^, Juan M. Alba^1^, Thomas Van Leeuwen^4^, Angelo Pallini^2^, Maurice W. Sabelis^1^†, Arne Janssen^1,2^*

^1^IBED, Department of Evolutionary and Population Ecology, University of Amsterdam, Science Park 904, 1098 XH Amsterdam, The Netherlands; ^2^Laboratório de Acarologia, Departamento de Entomologia, Universidade Federal de Viçosa, 36.570-000 Viçosa, MG, Brazil; ^3^ Present address: EPAMIG Centro Oeste, Prudente de Moraes, MG, Brazil; ^4^Department of Plants and Crops, Faculty of Bioscience Engineering, Department of Plants and Crops, Ghent University, Ghent, Belgium; ^5^ Present address: R&D Department, Biobest Group NV, Westerlo, Belgium

†Deceased

* Correspondence: Arne Janssen, Science Park 904, 1098 XH Amsterdam, The Netherlands. E-mail: [arne.janssen@uva.nl](mailto:Arne.Janssen@uva.nl), Orcid: 0000-0001-5075-5303

**S1.** *Prey preference and adult performance on mixed and single diets*

Differences in predation among all treatments (mixed diet and single diets) were analysed with a linear mixed effects model (LME, package nlme, (Pinheiro et al. 2020) of R, 2020) with treatment and time as fixed factors and the identity of the predatory mites as a random factor. In both cases, the blocks in which the experiment was performed were initially entered as a second random factor, but proved not significant, so were removed. Contrasts were assessed with the package emmeans (Lenth 2019).

Owing to a lack of high-quality prey, predators on a diet of *T. evansi* killed significantly more eggs of this species on the first day than predators on a mixed diet (Fig. S1). This difference disappeared, however, during subsequent days, resulting in a significant interaction of diet with time (Fig. S1, LME: Likelihood ratio = 13.9, *d.f.* = 1, *P* < 0.001). The numbers of *T. urticae* eggs killed also differed significantly through time (Fig. S1, LME: interaction of diet with time: Likelihood ratio = 17.6, *d.f.* = 1, *P* < 0.0001). On the first day, the numbers of eggs of *T. urticae* killed did not differ between predators on a diet of *T. urticae* and a mixed diet, but predators on a mixed diet killed significantly fewer *T. urticae* eggs on subsequent days (Fig. S1).

The average total numbers of eggs killed per individual also varied differentially through time for the three diets (Fig.S1, LME: Likelihood ratio = 16.9, *d.f.* = 2, *P* = 0.0002). On each day, predators on a diet of *T. evansi* eggs killed significantly fewer eggs than predators on other diets (Fig. S1). On the first day, predators with a mixed diet killed similar numbers of eggs as those with *T. urticae* eggs, but killed significantly fewer eggs on subsequent days (Fig. S1). The total numbers of eggs killed by predators with *T. evansi* eggs also decreased significantly from the first to the second day (Fig. S1).

**Fig. S1.** Average predation (± s.e. of the total) of adult female *P. persimilis* on diets of eggs of *T. urticae*, *T. evansi* or on a mixture of the two (vertical text along horizontal axis gives diet). Different letters above bars give significance of difference among diets per day, white capital letters within bars give significance of difference among days per diet (contrasts after LME, *P* < 0.05).

**S2.** *Reversibility of diet effects*

Data of the numbers of eggs killed by juvenile predators were analysed separately for the first two days (excluding immatures that did not receive any eggs, treatment “No Food” for obvious reasons) with an LME with time, diet and their interaction as factors and individual immature as random factor to correct for repeated measures. Because all individuals on a diet of *T. urticae* were adult by day 4, predation of all three treatment groups could only be compared on the third day, which was done with a GLM with a Poisson error distribution (log link) with diet as fixed factor. The predation during the last two days of the individuals that had received no food or *T. evansi* eggs was furthermore compared with an LME as above.

Juveniles that were offered *T. evansi* during the first two days killed significantly more eggs during the first day and significantly fewer eggs during the second day than individuals offered eggs of *T. urticae* (Fig. S2a, LME, interaction of time with diet: Likelihood ratio = 23.4, *d.f.* = 1, *P* < 0.0001). The numbers of eggs killed did not differ significantly during the third day, when all individuals were offered eggs of *T. urticae* (Fig. S2a, day 3, GLM: Chi^2^ = 3.64, d.f. = 2, *P* = 0.162), neither did it differ significantly during the 3^rd^ and 4^th^ day for individuals that had received no food or eggs of *T. evansi*. (Fig. S2a, LME: effect of previous diet: Likelihood ratio = 0.985 *d.f.* = 1, *P* = 0.321).

Predation data of adult predators that did not survive until the fourth day of the experiment were excluded from the analysis, as explained in the main text. The remaining data were analysed with an LME with individuals as a random factor and treatment and day as factors. To check whether the predators receiving the three different treatments did not differ from each other before treatments, we first analysed predation on the first two days for all three groups together. Subsequently, predation data of the third and the fourth day were analysed for the two groups that received prey, and for all three groups for the last two days.

During the first two days, when all adults received a diet of eggs of *T. urticae*, the numbers of prey eggs killed did not differ significantly among groups (Fig. S2b, LME, Likelihood ratio = 0.023, *d.f.* = 2, *P* = 0.989). During the second period, the group receiving eggs of *T. evansi* killed significantly fewer eggs than the group that continued receiving *T. urticae* eggs (Fig. S2b, LME: Likelihood ratio = 55.0, *d.f.* = 1, *P* < 0.0001). In the third period, when all predators received eggs of *T. urticae*, the numbers of eggs killed again did not differ significantly among the groups (Fig. S2b, LME, Likelihood ratio = 3.19, *d.f.* = 2, *P* = 0.20).

**Fig. S2.** The reversibility of the effect of a diet of *T. evansi* eggs (**a**) in juvenile predators and (**b**) in adults. **a.** Average predation rate of juveniles (± s.e.). One day old larvae, which soon developed into the next stage, were kept on a diet of eggs of *T. urticae*, *T. evansi* or without food for two days (grey background) and were subsequently reared to adulthood or until they died on a diet of *T. urticae* eggs. **b.** Average predation rate (± s.e.) of adult females that were offered a diet of *T. urticae* eggs during the first two days of the experiment. Subsequently, they received a diet of *T. urticae* or *T. evansi* eggs or no food during two days (grey background), after which all three groups were returned to a diet of *T. urticae* eggs during the last two days.

**References**

Lenth R (2019) emmeans: Estimated Marginal Means, aka Least-Squares Means. https://CRAN.R-project.org/package=emmeans

Pinheiro J, Bates D, DebRoy S, et al (2020) NLME: Linear and Nonlinear Mixed Effects Models. In: HttpCRANR-Proj. http://CRAN.R-project.org/package=nlme

R Core Team (2020) R: A language and environment for statistical computing. Version 4.0.2. R Foundation for Statistical Computing, Vienna, Austria. URL http://www.R-project.org
